# An anti-inflammatory and low fermentable oligo, di, and monosaccharides and polyols diet improved patient reported outcomes in fibromyalgia: A randomized controlled trial

**DOI:** 10.3389/fnut.2022.856216

**Published:** 2022-08-15

**Authors:** Ana Rita Silva, Alexandra Bernardo, Maria Fernanda de Mesquita, José Vaz-Patto, Pedro Moreira, Maria Leonor Silva, Patrícia Padrão

**Affiliations:** ^1^Faculdade de Ciências da Nutrição e Alimentação, Universidade do Porto, Porto, Portugal; ^2^Centro de Investigação Interdisciplinar Egas Moniz, Instituto Universitário Egas Moniz, Almada, Portugal; ^3^Instituto Português de Reumatologia, Lisbon, Portugal; ^4^EPIUnit, Instituto de Saúde Pública, Universidade do Porto, Porto, Portugal; ^5^Laboratório para a Investigação Integrativa e Translacional em Saúde Populacional, Porto, Portugal; ^6^Centro de Investigação em Atividade Física, Saúde e Lazer, Universidade do Porto, Porto, Portugal

**Keywords:** fibromyalgia, inflammation, anti-inflammatory diet, low FODMAPS diet, SIBO, small intestinal bacterial overgrowth, dysbiosis, randomized controlled trial

## Abstract

**Background:**

Fibromyalgia (FM) has been associated with dysbiosis and low-grade inflammation. Studies have reported that diet influences clinical features in FM.

**Objective:**

To evaluate the effect of an anti-inflammatory and low fermentable oligo, di, and monosaccharides and polyols (FODMAP) diet on clinical outcomes of patients with FM.

**Methods:**

This two arms Randomized Controlled Trial (NCT04007705) included 46 female patients with FM. The intervention group (*n* = 22) adopted an anti-inflammatory diet for 3 months, excluding gluten, dairy, added sugar, and ultra-processed foods, along with a low FODMAPs diet in the first month. The control group (*n* = 24) followed general healthy eating recommendations. Both diets were applied by a certified dietitian. Before and after the intervention, participants were assessed regarding pain, fatigue, gastrointestinal symptoms, quality of sleep, and quality of life, through the Revised Fibromyalgia Impact Questionnaire (FIQR), Visual Analogue Pain Scale (VAS), Visual Analog Scale from gastrointestinal symptoms (VAS GI), Brief Pain Inventory (BPI), Pittsburg Sleep Quality Index (PSQI), Fatigue Severity Survey (FSS), and The Short Form Health Survey (SF-36). A blood sample was collected and high-sensitive C-Reactive Protein and Erythrocyte Sedimentation Rate were quantified. Paired Samples *t*-test/Wilcoxon and independent samples *t*-test/Mann−Whitney were used to compare variables between groups.

**Results:**

After intervention, there was an improvement in intervention group scores of FIQR (*p* = 0.001), VAS (*p* = 0.002), BPI (*p* = 0.011), FSS (*p* = 0.042), VAS_GI (*p* = 0.002), PSQI (*p* = 0.048), and SF36 (*p* = 0.045) compared to control group. Inflammatory biomarkers (hs-CRP, ESR) did not change in both groups. The intervention was beneficial in the intervention group, regardless of age, disease duration, body mass index variation, and body fat change between baseline and post-intervention.

**Conclusion:**

An anti-inflammatory and low-FODMAP diet improved clinical features in patients with FM and may be useful as a complement to pharmacological therapy.

**Clinical Trial Registration:**

[https://clinicaltrials.gov/ct2/show/NCT04007705], identifier [NCT04007705].

## Introduction

Fibromyalgia (FM) is a chronic non-degenerative disease, characterized by generalized chronic musculoskeletal pain, fatigue, asthenia, anxiety, depression, changes in sleep patterns, and gastrointestinal symptoms similar to Irritable Bowel Syndrome (IBS) (1).

Fibromyalgia pathophysiology is still not known. However, low-grade inflammation is described by several authors, through a plasma pro-inflammatory cytokines increase, particularly interleukin (IL)-6 and IL-8 (2, 3). Literature suggests that saturated fatty acids (SFA), *trans* fatty acids, and cholesterol intake, included in the “Dietary Inflammatory Index” (4), together with gluten (5), dairy products (6), and ultra-processed foods (7), could have a pro-inflammatory effect. On the other hand, it is known the anti-inflammatory potential of mono- and poly-unsaturated fatty acids (PUFA) (4), specially omega-3 (8), and antioxidants compounds in the diet (9).

Furthermore, several studies showed an association between FM and dysbiosis (10), and in particular, with Small Intestinal Bacterial Overgrowth (SIBO) (11, 12), characterized by the inappropriate colonization of the distal small bowel with colonic bacteria (12). SIBO is usually treated with antibiotics (13). However, the association between antibiotic use and abuse with the development of dysbiosis is well known (14, 15). Dietary therapy, specifically a 4-week low fermentable oligo, di- and monosaccharides and polyols (FODMAPs) diet protocol, seems to be a complementary approach to SIBO treatment, through the exclusion of rapidly fermentable, short-chain carbohydrates (16).

As pharmacological therapy seems not to completely resolve the symptoms of the disease (1), a dietary intervention that includes potentially anti-inflammatory foods and excludes the potentially pro-inflammatory ones, and that simultaneously allows an optimization of the intestinal microbiota, emerges as an opportunity to improve the FM patient’s reported outcomes. In a recent study, it was reported that pain and functional repercussions in FM, along with the quality of life, quality of sleep, anxiety, depression, and inflammatory biomarkers, seem to improve with a hypocaloric diet, a raw vegetarian diet, or a low FODMAPs diet. However, the existing clinical trials on this subject are scarce and of low quality, which does not allow conclusions to be drawn (17). Additionally, to our knowledge, a dietary approach involving a combination of several anti-inflammatory dietary factors has never been designed. Therefore, the aim of this study is to evaluate the effect of a potent anti-inflammatory and low FODMAPs diet on clinical features, namely pain, fatigue, sleep quality, gastrointestinal alterations, and inflammatory biomarkers of patients with FM.

## Materials and methods

The detailed study protocol of this Randomized Controlled Clinical Trial (RCT) has been published elsewhere (18) and registered on Clinicaltrials.gov with the identification number: NCT04007705.

### Ethical considerations

This study was approved by the Ethics Committee of the Portuguese Institute of Rheumatology, with reference number 4/2020, and was carried out in accordance with the Declaration of Helsinki (Declaration of 1975, revised in 2000). Informed consent was given to all participants, after oral and written information about the study.

### Study design and participants

This parallel-group RCT with two arms took place between April 2019 and June 2020 at the Portuguese Institute of Rheumatology (*Instituto Português de Reumatologia*) in Lisbon, Portugal.

Since FM affects mainly women, it has been decided to recruit only women for the present study, in order to improve the statistical power. Forty-six female adults, aged between 18 and 75 years old, which were not currently undergoing lactation or pregnancy, and with the ability to read and sign the informed consent were eligible to integrate the study. FM diagnosis has been performed by a Rheumatologist, according to the Rome III criteria of the American College of Rheumatology, revised in 2010 (19), with a stable dose therapy within 4 weeks before the study begins.

Patients with the presence of other inflammatory diseases or uncontrolled medical conditions (e.g., Diabetes Mellitus, heart disease, renal failure, neoplastic diseases, and liver diseases), with a prior or current clinical history of abuse of the drug or other substances, or with diagnose of any pathologies that prevent to follow the dietary intervention identified by the physician were not included. Patients which changed pharmacological therapy during the intervention period were excluded.

After eligibility criteria, confirmation, and informed consent were signed, participants were randomly allocated to an intervention or control group. Simple randomization using a random sequence was applied to the first participant who enrolled in the study. The remaining patients were systematically allocated to intervention (G1) or control group (G2), as they were recruited. Each participant was given a code and anonymity and confidentiality of the collected data were assured.

Sixty-two patients were assessed for eligibility, 61 were included and 46 completed the study. For 3 months, the intervention group adopted a two phases intervention: the first phase, occurred in the first month, in which a combined anti-inflammatory diet and low FODMAPS diet were adopted; the second phase occurred in the second and third subsequent months, in which participants continued only with the anti-inflammatory diet. The Control group adopted a healthy diet, based on the World Health Organization (WHO) general recommendations (20). Both dietary interventions were implemented by a certified dietitian, which give the instructions, monitored compliance, and followed up with the participants.

Patient Reported Outcomes were collected by interview using structured validated questionnaires, and a blood sample was taken for the measurement of serum inflammatory biomarkers, before and after the intervention.

Patients were monitored through biweekly telephone contacts, being also possible for the patient to clarify any question through the contact provided. Physical activity, medical therapy, and supplements were monitored in every contact, in order to ensure no changes, which would imply patient exclusion. A 3-day food dairy was also applied in every contact, to ensure patient compliance to the diet, which would also imply the exclusion.

### Dietary implementation

#### Intervention group

The intervention group adopted an anti-inflammatory diet, excluding potential inflammatory components/foods, such as gluten, dairy products, free sugars, and ultra-processed food. Furthermore, the ingestion of foods rich in omega-3 fatty acids, antioxidants, and dietary fiber was promoted, according to the “Dietary Inflammatory Index” (4, 21). During the first month of intervention, a low FODMAPs diet criteria have been added to the anti-inflammatory diet, with the exclusion of foods rich in sugars more fermentable by bacteria. After the first month of intervention, all fruit and vegetables previously excluded were reintroduced, keeping the anti-inflammatory diet for another 2 months, completing a total of 3 months of intervention. A trained certified dietitian provided recipes in order to help diet compliance.

It was envisaged that the low FODMAPs protocol would provide an improvement in patients’ gastrointestinal symptoms. Additionally, it was intended to evaluate the effects of the anti-inflammatory diet on the remaining symptoms of patients with FM.

The integration of the two dietary strategies in global nutritional intervention, rather than an individualized one, may have a greater and more consistent effect in the relief of FM symptoms, compared to the restriction of isolated dietary components, which is defended by many authors (22, 23).

#### Anti-inflammatory diet

The anti-inflammatory diet combined the exclusion of potentially pro-inflammatory components and the inclusion of potentially anti-inflammatory ones.

Gliadin, present in gluten, is one of the known causes of intestinal hyperpermeability, which triggers an immunological reaction of inflammatory character (24), described by several authors as low-grade inflammation (25). Dairy was excluded considering the variation of beta-casein genotypes in milk and their possible association with gastrointestinal symptoms (26) and increased intestinal inflammation through activation of the Th2 signaling pathway in the intestine (27). Sugar has a recognized inflammatory activity, as its excessive consumption promotes the production of free radicals, leading to an increase in oxidative stress (28, 29). Many ultra-processed foods are considered potentially inflammatory due to their free sugars, hydrogenated fat, and food additives content (30, 31). Additionally, it is known that its relevant accumulation of Advanced Glycation End-products (AGEs) is also related to a pro-inflammatory effect (32, 33), by promoting TNFα, IL6, VCAM1, Th1, Treg, Th2, and Th17 liberation, which induce inflammation (34, 35).

On the other hand, to increase antioxidant and anti-inflammatory potential, the ingestion of 3 pieces of fruit a day and half a plate of vegetables two times a day was promoted. The intake of red fruits, strawberries, pomegranates, red grapes, apple (rich in flavonols, such as resveratrol and quercetin), orange, kiwi, and papaya (rich in vitamin C) was indicated. The intake of indole-3-carbinol and sulforaphanes present in broccoli, cauliflower, and cabbage was promoted, with the indication of cooking for a maximum of 5 min to preserve it. It also promoted the increased intake of beta-carotene rich foods (carrots, pumpkins, orange sweet potatoes), lycopene (tomatoes, blueberries), gingerol (ginger), and catechins (cocoa and green tea) (36). Moreover, it is well known the omega-3 anti-inflammatory capacity, especially at an adequate omega-6:omega-3 ratio. It allows the production of prostaglandins, leukotrienes, resolvins, and protectins, promoting the expression of anti-inflammatory cytokines (21, 37). Therefore, the consumption of omega-3 rich food such as salmon, tuna, mackerel, and sardines, as well as walnuts, almonds, and linseeds, was promoted. Furthermore, the replacement of sunflower oil, butter, and margarine for extra virgin olive oil was also indicated, for an increase in monounsaturated fatty acids and a reduction in omega-6 and saturated fat. Additionally, the maintenance of blood glucose homeostasis was promoted, through an adequate intake of dietary fiber, protein and fat, and a balanced intake of carbohydrates, since is one of the most important factors in an anti-inflammatory diet.

### Low fermentable oligo, di, and monosaccharides and polyols diet

The low FODMAPs diet is characterized by the avoidance of rapidly fermentable, short-chain carbohydrates, namely monosaccharides (fructose), disaccharides (lactose), oligosaccharides (fructans and galactans), and polyols (sorbitol, mannitol, xylitol, and maltitol). These sugars have a slower absorption rate, due to a reduced transport capacity across the epithelium or by the inhibition of the activity of brush border enzymes disaccharidase and hydrolase, which makes them more susceptible to being fermented by bacteria present in the intestine (16, 38). The protocol requires the exclusion of lactose-containing dairies; all cereals except rice and oat; cashew; all fruits other than banana, citrus, pineapple, red berries, strawberries, and kiwi; and all vegetables other than pumpkin, eggplant, green beans, celery, cabbage, lettuce, tomato, capsicum, carrot, and cucumber (16). The food reintroduction should be carried out progressively and individually, taking into account the existence of possible food intolerances. However, given the nature of the study, it was not possible to gradually reintroduce the excluded food. Consequently, in the second and third months of the intervention, all vegetables, legumes, and fruits were reintroduced, and participants continued only with the anti-inflammatory diet.

The presence of dysbiosis (39–41), and in particular SIBO (11, 12) has been described in patients with FM, with a significant improvement in pain, fatigue, gastric pain, mobility, and gastrointestinal symptoms, after 4 weeks of low FODMAPs diet (42). Marsh et al’s (11) meta-analysis support the efficacy of a diet with a low intake of foods rich in FODMAPs for 4−6 weeks in the treatment of gastrointestinal symptoms, including abdominal pain, abdominal distention, constipation, diarrhea, and flatulence.

### Control group

The control group adopted healthy eating WHO recommendations which were explained to participants. According to WHO, a healthy diet contains at least 400 g of fruits and vegetables, other than potatoes, sweet potatoes, cassava, and starchy roots. Consumption of legumes, nuts, and whole grains (wheat, maize, millet, oats, rice, rye), was also promoted, as well as an intake of less than 5 g of salt per day, less than 10% of total energy intake from free sugars and less than 30% of total energy intake from fats, giving preference to unsaturated fats (20).

### Socio-demographic and lifestyle characteristics assessment

Socio-demographic characteristics of the patients were collected, namely age, education level (<9 schooling years or ≥9 schooling years), and work status (employed, unemployed, retired, or domestic/pensioner).

Life-style characteristics, such as smoking habits (recoded as smoker or non-smoker), frequency of alcohol beverages intake (recoded as daily or occasionally, since only one participant reported a regular consumption), and structured physical exercise (<1 h a week or ≥1 h a week), were collected. Additionally, it was also registered the disease duration and usual pharmacological therapy.

### Anthropometric and body composition assessment

Data on anthropometric measurements namely waist circumference, height, and weight were assessed at the beginning and the end of the intervention. Body mass index (BMI) (kg/m^2^) was calculated, and WHO classification was used to categorize BMI (43).

Body composition parameters namely fat mass percentage, muscular mass, and total body water were estimated by bio-impedance, through the scale Inbody^®^, model 770.

Post-intervention and baseline differences were arithmetically calculated for each anthropometric and body composition variable.

### Patient reported outcomes

The primary Patient Reported Outcomes of interest for this study were pain, fatigue, quality of sleep, quality of life, and gastrointestinal symptoms, which were assessed through specific questionnaires.

Revised Fibromyalgia Impact Questionnaire (FIQR) (44), was used to assess the impact of FM on the patient’s life. It consists of 21 questions that evaluate clinical severity, health status, and ability to daily activities of patients with FM. A score between 0 and 100 is obtained, which is lower as the quality of life improves.

Visual Analogue Pain Scale (VAS) (45) and Brief Pain Inventory (BPI) were used to assess pain (46). VAS is a one-item questionnaire about pain, which score range is between 0 and 10, being 0 equivalent to no pain and 10 the worst pain ever felt. BPI measures pain intensity and pain interference in daily activities. The score ranges between 0 and 20, being lower as lower pain is felt.

To assess gastrointestinal symptoms, the Visual Analog Scale from a list of common gastrointestinal and extraintestinal symptoms in FM, IBS, and Non-Celiac Gluten Sensitivity (VAS_GI) (47) was applied. VS._GI score was between 0 and 10, being 0 equivalent to very good gastrointestinal function and 10 to very bad gastrointestinal function.

Fatigue Severity Survey (FSS) (48) was used to assess the fatigue level. This tool is a 9 items questionnaire that evaluates motor aspects of fatigue and its impact on individual’s daily functioning. The scale ranges from 0 to 7 and reveals less fatigue the lower the score obtained.

Pittsburg Sleep Quality Index (PSQI) (49) was used to assess the quality of sleep. This questionnaire evaluates subjective sleep quality, sleep latency, sleep duration, habitual sleep efficiency, sleep disturbance, use of sleeping pills, and daytime dysfunction. PSQI score range is between 0 and 21. A total score above 5 indicates poor sleep quality.

To assess the quality of life, Short Form 36 (SF-36) (50) was used. SF-36 is a 36 items tool that focuses on general health, physical functioning, vitality, physical pain, mental health, social functioning, and emotional impact on daily tasks. The score range is between 0 and 100, being 100 equivalent to the better possible quality of life. It encompasses both Mental and Physical Health that were quantified separately, in addition to the whole questionnaire.

### Biochemical parameters assessment

A blood sample was collected at baseline and post-intervention. Blood tests were carried out by analysts from *Joaquim Chaves Saúde* Laboratory, at the Portuguese Institute of Rheumatology. Serum high-sensitive C-Reactive Protein (hs-CRP) and Erythrocyte Sedimentation Rate (ESR) were measured through immunoturbidimetry (51) and the Westergren method (52) respectively, to assess the presence of inflammation. Despite being both non-specific markers, the combination of the two allows obtaining information on the individual’s inflammatory phenotype. Being an acute phase protein, CRP reveals the presence of inflammation in its initial phase, increasing after 4−6 h. On the other hand, the ESR increases within 24−48 h and gradually decreases, allowing for the assessment of the response to treatment (53).

### Dietary assessment

At baseline, a 24-h dietary recall was applied to verify the homogeneity of dietary intake between groups. Every biweekly telephone contact and at the end of the intervention, a 3-day food record was completed by each participant in order to ensure the intervention compliance. Study participants were carefully instructed by a dietitian to complete the food record. If necessary, participants estimated the food amounts with a picture book which estimate the portion sizes for meals (54).

The Food Processor^®^ software version 11.2.274 was used to convert food into nutrients. Energy and nutrients were expressed by average values calculated from the 3-day food records. Protein, carbohydrates, of which sugars, monosaccharides, disaccharides, and added sugars, total fat, of which MUFA, PUFA, omega-3, and omega-6 were expressed by percentage of TEI (% TEI). Dietary fiber was expressed in grams and g/1,000 kcal.

Additionally, the average 3-day food record of the ingested amount of food containing gluten in its composition (bread, biscuits, cake, pasta, savory, breakfast cereals, cereal bars) was manually collected from food diaries and 24 h report. The same foods in the gluten-free version were not considered. Moreover, dairy products (milk, yogurt, cheese, butter), ultra-processed products according to the NOVA classification system (55), and sugar added to beverages were also collected and expressed in grams.

### Statistical analysis

Statistical analysis was performed using IBM SPSS Statistics Software, version 19.0.

Descriptive data were presented as mean, standard deviation (SD), median, percentile (P) 25 and P75 for continuous variables, or the frequency (number and percentage) for categorical variables.

To compare FM symptoms and inflammatory biomarkers within groups at baseline and post-intervention, Paired Samples *t*-tests or Wilcoxon signed rank tests were used for continuous variables, as appropriate.

Independent Samples *t*-test or Mann−Whitney *U*-test was used to compare FM symptoms, inflammatory biomarkers, and dietary intake between groups at baseline and post-intervention moments, as appropriate. The arithmetic differences between baseline and post-intervention were calculated for dietary intake and clinical features for each group. MANOVA was applied to assess the effect of the intervention between groups.

Additionally, a General Linear Model (GLM) was used in order to assess the impact of the intervention by adjusting for potential confounders, namely age, disease duration, variation of BMI, and variation of body fat percentage. GLM was also used to verify the possible isolated effect of each nutrient and food with anti-inflammatory potential in the clinical features.

In order to define the sample size required for the study, and to give a statistical power of 80%, G-Power Software version 3.1.9.4 revealed that, for a desirable effect size of 50%, a minimum sample size of 45 individuals was required. The sample size was calculated with respect to primary outcomes, namely pain and fatigue.

## Results

The effect of an anti-inflammatory and low fermentable oligo, di- and monosaccharides and polyols (FODMAP) diet on clinical outcomes of FM patients are presented in this section.

### Baseline characteristics of the participants

The study sample consisted of 62 adult female patients with FM, of which 46 patients completed the study. There were no significant differences between intervention group (*n* = 22) and control group (*n* = 24) for demographics, life-style characteristics and body composition (Table 1).

**TABLE 1:** Baseline socio-demographic and lifestyle characteristics of the participants.

Characteristics	Control group^1^ (*n* = 24) mean (±SD) median (P25;P75)	Intervention group^1^ (*n* = 22) mean (±SD) median (P25;P75)	*P*-value
Age (years)	56 (±8) 57 (51; 59)	60 (±6) 60 (56; 66)	0.057^a^
Disease duration (years)	13 (±9) 13 (4; 20)	14 (±8) 17 (5; 20)	0.526^a^
**Body mass and composition**			
Body mass index (kg/m^2^)	30 (±6) 29 (26; 34)	29 (±4) 29 (25; 31)	0.531^a^
Waist circumference (cm)	99 (±14) 101 (90; 109)	98 (±10) 101 (89; 106)	0.783^a^
Fat mass (%)	39 (±9) 41 (33; 44)	39 (±6) 38 (34; 44)	0.796^a^
Muscle mass (kg)	24 (±3) 24 (21; 27)	23 (±2) 23 (21; 25)	0.502^b^
Total body water (%)	45 (±7) 43 (41; 50)	45 (±6) 45 (41; 47)	0.758^b^
	***n*** **(%)**	***n*** **(%)**	
**Education (schooling)**			
<9 years	14 (60.9)	10 (45.5)	0.388
≥9 years	9 (39.1)	12 (54.5)	0.152
**Work status**			
Employed	10 (43.5)	8 (36.4)	0.541
Unemployed	3 (13.0)	1 (4.5)	0.344
Retired	5 (21.7)	8 (36.4)	0.248
Domestic/pensioner	5 (21.7)	5 (22.7)	0.327
**Smoking habits**			
Smoker	2 (8.7)	3 (13.6)	0.568
Non-smoker	21 (91.3)	19 (86.4)	0.777
**Alcoholic beverages consumption**			
Daily	2 (8.7)	0 (0)	0.338
Occasional/Never	21 (91.3)	22 (100)	0.090
**Exercise frequency**			
<1 h/week	22 (91.7)	18 (81.8)	0.596
≥1 h/week	2 (8.3)	4 (18.2)	0.823

^1^Female participants.

SD, standard deviation; P25, percentile 25; P75, percentile 75.

^a^P-value calculated by Independent-Samples T-Test between control and intervention groups mean values;

^b^P-value calculated by Mann−Whitney Test between control and intervention groups mean values.

Almost 40% of the participants were employed and had less than 9 schooling years. More than 85% reported being non-smokers, more than 91% did not drink alcoholic beverages daily and more than 91% exercised less than 1 h a week. Both groups had a body fat mass average of 39%, and a BMI of nearly 30 kg/m^2^.

Regarding usual pharmacological treatment, over 50% in both groups were medicated with analgesics and muscle relaxants, and approximately 75% reported taking antidepressants, anxiolytics, or sedatives. Pharmacologic therapy and supplementation that were already being performed by the participants, namely vitamin D, magnesium, and calcium, were not changed throughout the intervention.

### Dietary parameters

At baseline, no significant differences were observed between groups in most of the nutritional parameters, except for the intake of total energy and omega-3 fatty acids, and for the consumption of added sugars and ultra-processed products which were significantly higher in the control group (Tables 2A,B, 3).

**TABLE 2A:** Dietary intake in control and intervention group at baseline^1^ and post-intervention^2^.

Outcomes	Control group (*n* = 24)^3^				Intervention group (*n* = 22)^3^			
		
	Baseline mean (±SD) median (P25;P75)	Post-intervention mean (±SD) median (P25;P75)	Post-intervention–baseline difference Δ mean (±SD) Δ median (P25; P75)	Within-group analysis *p*-value	Baseline mean (±SD) median (P25;P75)	Post-intervention mean (±SD) median (P25;P75)	Post-intervention–baseline difference Δ mean (±SD) Δ median (P25; P75)	Within-group analysis *p*-value
Total energy intake (kcal)	1773 (±374) 1710 (1488; 2030)	1725 (±374) 1722 (1397; 1976)	−48.3 (±446.5) 68.7 (420.1; 229.4)	0.775^b^	1471 (±362) 1455 (1255; 1736)	1256 (±355) 1320 (1176; 1403)	−195.8 (±544) −13.9 (−412.3; 140.5)	0.236^b^
Protein (% TEI)	20 (±5) 19 (17; 23)	19 (±3) 20 (17; 22)	−1.2 (±4.9) −0.8 (−4.0; 2.2)	0.246^a^	21 (±4) 21 (19; 24)	19 (±3) 18 (17; 22)	−2.1 (±4.2) −1.6 (−4.8; 0.4)	0.030^a^
Carbohydrate (% TEI)	49 (±8) 50 (45; 55)	49 (±5) 50 (46; 52)	0.4 (±6.4) −0.2 (−4.2;3.9)	0.767^a^	51 (±9) 53 (44; 57)	46 (±6) 46 (41; 49)	−5.9 (±9.9) −4.9 (13.2; 1.4)	0.011^a^
Sugars (% TEI)	19.7 (±7.1) 20.0 (13.5; 23.8)	14.0 (±7.7) 15.2 (8.1; 19.5)	−5.7 (±8.9) −4.8 (−8.7; −0.7)	0.005^a^	19.8 (±7.6) 18.3 (14.2; 26.4)	12.3 (±7.1) 14.2 (9.2; 15.6)	−7.5 (±9.1) −7.6 (−14.6; −1.3)	0.001^a^
Monosaccharides (% TEI)	5.0 (±2.3) 4.7 (2.9; 7.2)	5.2 (±2.9) 4.9 (3.2; 6.9)	0.2 (±3.3) 0.4 (−2.4; 2.9)	0.821^a^	4.9 (±2.9) 4.6 (2.6; 6.3)	5.5 (±3.4) 5.8 (3.6; 7.6)	−0.5 (±4.1) 0.9 (−2.4; 2.7)	0.542^a^
Disaccharides (% TEI)	4.6 (±3.0) 4.2 (2.1; 7.0)	4.2 (±2.5) 3.9 (2.6; 5.6)	0.4 (±2.6) 0.2 (−1.6; 1.8)	0.440^a^	4.9 (±2.6) 5.2 (2.6; 6.6)	1.7 (±1.3) 1.5 (0.9; 2.7)	−3.3 (±3.0) −3.5 (5.1; 0.9)	*p* < 0.001^a^
Added sugars (% TEI)	0.8 (±1.6) 0.0 (0.0; 1.7)	0.7 (±0.9) 0.0 (0.0; 1.4)	−0.1 (±1.3) 0.0 (0.0; 0.3)	0.386^b^	0.5 (±1.4) 0.0 (0.0; 0.0)	0.0 (±0.0) 0.0 (0.0; 0.0)	−0.5 (±1.5) 0.0 (0.0; 0.0)	0.144^b^
Dietary fiber (g)	17.9 (±3.7) 17.7 (14.9; 20.3)	17.0 (±7.6) 18.3 (11.5; 21.7)	−0.9 (±8.1) 0.9 (−6.6; 3.6)	0.710^b^	16.0 (±5.6) 16.4 (10.7; 19.9)	16.5 (±9.5) 17.5 (13.6; 21.6)	0.5 (±11.5) 0.9 (−6.8; 7.3)	0.858^b^
Dietary fiber (g/1000 kcal)	1.7 (±0.4) 1.8 (1.5; 2.0)	1.9 (±0.5) 1.8 (1.3; 2.2)	0.1 (±0.6) 0.2 (−0.4; 0.4)	0.680^a^	1.6 (±0.6) 1.6 (1.0; 1.9)	2.0 (±0.6) 1.8 (1.6; 2.5)	0.4 (±0.9) 0.4 (−0.3; 0.8)	0.037^a^

^1^Values refer to 24h prior first contact (at baseline).

^2^Values are the average of the 3 days prior to the date of post intervention.

^3^ Amount of food containing gluten in its composition (bread, biscuits, cake, pasta, savory, breakfast cereals, cereal bars).

^4^Female participants. SFA = Saturated Fatty Acid; MUFA = Monounsaturated fatty acid; PUFA = Polyunsaturated fatty acid; n-3 = Omega 3 Fatty Acid; n-6 = Omega 6 Fatty Acid; TEI = Total Energy Intake.

^a^p-value calculated by Paired Samples T-Test between baseline and post-intervention, within-groups mean values;

^b^p-value calculated by Wilcoxon Test between baseline and post-intervention, within-groups mean values.

**TABLE 2B:** Dietary intake in control and intervention group at baseline^1^ and post-intervention^2^.

Outcomes	Control group (*n* = 24)^3^				Intervention group (*n* = 22)^3^			
		
	Baseline mean (±SD) median (P25;P75)	Post-intervention mean (±SD) median (P25;P75)	Post-intervention–baseline difference Δ mean (±SD) Δ median (P25; P75)	Within-group analysis *p*-value	Baseline mean (±SD) median (P25;P75)	Post-intervention mean (±SD) median (P25;P75)	Post-intervention–baseline difference Δ mean (±SD) Δ median (P25; P75)	Within-group analysis *p*-value
Total fat (% TEI)	30 (±6) 30 (26; 34)	31 (±6) 31 (27; 35)	1.2 (±7.4) −0.1 (−3.4; 7.9)	0.407^b^	28 (±8) 27 (23; 29)	37 (±7) 37 (32; 41)	9.4 (±9.8) 10.5 (0.6; 14.5)	0.001^b^
SFA (% TEI)	8.2 (±2.1) 8.3 (6.4; 10.4)	7.3 (±3.0) 7.5 (6.0; 10.1)	−0.9 (±2.9) 0.1 (−2.3; 0.9)	0.440^b^	7.8 (±2.5) 7.6 (6.2; 10.1)	4.9 (±2.7) 5.7 (3.6; 6.9)	−3.0 (±4.1) −2.3 (−6.6; 0.2)	0.006^b^
MUFA (% TEI)	5.7 (±2.5) 5.5 (4.2; 6.6)	4.8 (±2.1) 5.1 (3.9; 5.8)	−0.9 (±3.9) −0.3 (−2.1; 0.9)	0.331^b^	4.6 (±1.8) 4.6 (3.3; 5.4)	6.6 (±4.9) 5.9 (3.3; 9.0)	1.9 (±5.6) −0.8 (−1.7; 6.1)	0.123^b^
PUFA (% TEI)	13.0 (±4.5) 12.9 (10.2; 15.0)	13.9 (±5.2) 13.8 (11.9; 17.5)	0.8 (±7.3) 1.6 (−2.2; 5.9)	0.278^b^	11.2 (±5.2) 11.1 (7.3; 13.7)	16.2 (±8.4) 19.3 (14.4; 21.1)	5.0 (±10.1) 7.9 (1.2; 13.2)	0.022^b^
n-3 (% TEI)	0.9 (±0.6) 0.8 (0.5; 1.0)	0.9 (±0.5) 0.7 (0.6; 1.2)	−0.1 (±0.9) 0.1 (−0.2; 0.3)	0.530^b^	0.6 (±0.4) 0.4 (0.3; 0.6)	1.3 (±0.9) 1.3 (0.4; 1.9)	0.7 (±1.4) 0.4 (−0.4; 1.6)	0.046^b^
n-6 (% TEI)	4.5 (±1.8) 4.2 (3.5; 5.1)	3.9 (±1.8) 4.1 (3.0; 4.8)	−0.7 (±2.9) −0.3 (1.6; 0.9)	0.317^b^	3.7 (±1.7) 3.6 (2.6; 4.2)	5.1 (3.9) 4.6 (2.5; 8.2)	1.4 (±4.5) 0.9 (−1.8; 5.6)	0.149^b^
Food containing gluten^3^ (g)	179.8 (±92.4) 187.5 (105; 260)	150.9 (±54.9) 153.3 (116.3; 185)	−28.9 (±86.9) −14.2 (66.7; 28.3)	0.118^a^	170.6 (±71.8) 162.5 (118.8; 205)	0 0	−170.6 (±71.7) −162.5 (−205.0; −118.8)	*p* < 0.001^b^
Dairy products (g)	303.1 (±210) 234.6 (131.3; 501.3)	254.3 (±216.3) 235 (55.4; 358.7)	−48.8 (±150.9) 150.9 (−144.8; 54.2)	0.127^a^	290.2 (±220.3) 220.0 (138.3; 411.3)	0 0	−290.2 (±220.3) −220.0 (−411.3; −138.8)	*p* < 0.001^b^
Ultra-processed foods (g)	82.4 (±67.5) 67.2 (26.3; 142.5)	52.5 (±47.3) 47 (5.0; 78.8)	−29.9 (±78.6) −5.2 (−99.8; 17.5)	0.075^a^	47.3 (±44.1) 47.5 (0.0; 7.5)	0 0	−47.3 (±44.1) −47.5 (−75.0; 0.0)	*p* < 0.001^b^
Sugar added to foods (g)	4.0 (±0.0) 6.34 (0.0; 8.0)	3.0 (±4.5) 0 (0; 8)	−1.0 (±4.3) 0 (0; 0)	0.257^b^	1.1 (±3.7) 0 (0; 0)	0 0	−1.1 (±3.7) 0 (0; 0)	*p* < 0.001^b^

^1^Values refer to 24 h prior to first contact (at baseline).

^2^Values are the average of the 3 days prior to the date of post intervention.

^3^Amount of food containing gluten in its composition (bread, biscuits, cake, pasta, savory, breakfast cereals, cereal bars).

^4^Female participants. SFA = Saturated Fatty Acid; MUFA = Monounsaturated fatty acid; PUFA = Polyunsaturated fatty acid; n-3 = Omega 3 Fatty Acid; n-6 = Omega 6 Fatty Acid; TEI = Total Energy Intake.

^a^p-value calculated by Paired Samples t-test between baseline and post-intervention, within-groups mean values;

^b^p-value calculated by Wilcoxon Test between baseline and post-intervention, within-groups mean values.

**TABLE 3:** Dietary intake between control and intervention group analysis at baseline^1^ and post-intervention^2^.

Outcomes	Between-group analysis	
	
	Baseline *p*-value	Post-intervention *p*-value
Total energy intake (kcal)	0.008^b^	*p* < 0.001^b^
Protein (% TEI)	0.657^b^	0.777^a^
Carbohydrate (% TEI)	0.385^b^	0.049^a^
Sugars (% TEI)	0.981^b^	0.416^a^
Monosaccharides (% TEI)	0.885^b^	0.778^a^
Disaccharides (% TEI)	0.737^b^	0.001^a^
Added sugars (% TEI)	0.186^a^	0.003^b^
Dietary fiber (g)	0.235^a^	0.930^b^
Dietary fiber (g/1000 kcal)	0.169^b^	0.343^a^
Total fat (% TEI)	0.071^a^	0.004^a^
SFA (% TEI)	0.716^b^	0.004^b^
MUFA (% TEI)	0.062^a^	0.117^a^
PUFA (% TEI)	0.206^b^	0.018^b^
n-3 (% TEI)	0.006^a^	0.538^b^
n-6 (% TEI)	0.129^b^	0.391^b^
Food containing gluten^3^ (g)	0.707^a^	*p* < 0.001^b^
Dairy products (g)	0.848^a^	*p* < 0.001^b^
Ultra-processed foods (g)	0.044^a^	*p* < 0.001^b^
Sugar added to foods (g)	0.038^b^	*p* < 0.001^b^

^1^Values refer to 24 h prior to first contact (at baseline).

^2^Values are the average of the 3 days prior to the date of post intervention.

^3^Amount of food containing gluten in its composition (bread, biscuits, cake, pasta, savory, breakfast cereals, cereal bars) SFA = Saturated Fatty Acid; MUFA = Monounsaturated fatty acid; PUFA = Polyunsaturated fatty acid; n-3 = Omega 3 Fatty Acid; n-6 = Omega 6 Fatty Acid; TEI = Total Energy Intake.

^a^p-value calculated by Independent-Samples t-test between control and intervention groups mean values;

^b^p-value calculated by Mann−Whitney Test between control and intervention groups mean values.

In Tables 2A,B, 3 we may observe that only sugar ingestion lowered at the end of the 3 months. However, the intervention group reported significant changes after the implementation of the dietary protocol, with a negative variation in the contribution to TEI for protein (−2.1 ± 4.2% to TEI, *p* = 0.03), carbohydrates (−5.9 ± 9.9% to TEI, *p* = 0.011), sugars (−7.5 ± 9.1% to TEI, *p* = 0.001), disaccharides (−3.3 ± 3.0% to TEI, *p* < 0.001) and SFA (−3.0 ± 4.1% to TEI, *p* = 0.006). On the contrary, a positive variation was found for total fat (9.4 ± 9.8% to TEI, *p* = 0.001), PUFA (5.0 ± 10.1% to TEI, *p* = 0.022), omega-3 fatty acids (0.7 ± 0.046), and fiber/1,000 kcal (0.4 ± 0.9% to TEI, *p* = 0.037). Additionally, the intervention group reported the exclusion of sugar added to foods (baseline 1.1 ± 3.7 g; post-intervention 0 g, *p* < 0.001) and ultra-processed foods (baseline 47.3 ± 44.1 g; post-intervention 0 g, *p* < 0.001), as prescribed.

Despite the statistically similar baseline values, there were significant differences between the intervention and control groups in the post-intervention period regarding the intake of disaccharides, added sugar, and SFA, which was higher in the control group and concerning the intake of total fat and PUFA that was higher in the intervention group (Tables 2A,B, 3).

### Fibromyalgia clinical features

The differences between post-intervention and baseline showed significantly more favorable outcomes for the majority of parameters in the intervention group compared to the control group. Significantly greater improvement was found in FM severity scale FIQR in the intervention group compared to the control group (−19.9 ± 18.8 vs. −2.2 ± 16.1; *p* = 0.001). Significantly greater improvement was found in pain in intervention group compared to control group, both in VAS (−2.3 ± 2.5 vs. −0.04 ± 2.1; *p* = 0.002) and BPI questionnaires (−3.8 ± 4.1 vs. −1.1 ± 2.6; *p* = 0.011). Significantly greater improvement was found in gastrointestinal symptoms, through the VAS_GI questionnaire, in the intervention group compared to the control group (−2.0 ± 0.9 vs. −0.9 ± 1.3; *p* = 0.002). Significantly greater improvement was found in sleep quality, in the PSQI questionnaire, in the intervention group compared to the control group (−3.5 ± 4.6 vs. −1.2 ± 2.6; *p* = 0.048). Significantly greater improvement was found in fatigue, through the FSS questionnaire, in the intervention group compared to the control group (−1.1 ± 1.2 vs. −0.5 ± 1.0; *p* = 0.042). Significantly greater improvement was found in the quality of life, evaluated through SF36, in the intervention group compared to the control group (10.2 ± 11.2 vs. 3.6 ± 10.4; *p* = 0.045), specifically in the physical component (18.1 ± 20.0 vs. 3.9 ± 13.5; *p* = 0.008). SF36 score is higher as the quality of life improves (Tables 4, 5).

**TABLE 4:** Clinical features in control and intervention groups at baseline and post-intervention.

Outcomes	Control group (*n* = 24)^1^				Intervention group (*n* = 22)^1^			
		
	Baseline mean (±SD) median (P25;P75)	Post-intervention mean (±SD) median (P25;P75)	Post-intervention− baseline difference Δ mean (±SD) Δ median (P25; P75)	Within-group analysis *p*-value	Baseline mean (±SD) median (P25;P75)	Post-intervention mean (±SD) median (P25;P75)	Post-intervention− baseline difference Δ mean (±SD) Δ median (P25; P75)	Within-group analysis *p*-value
FIQR (Range: 0−100)	60.2 (±10.5) 60.5 (52.5; 68.9)	57.6 (±15.6) 61.2 (50.4; 68.4)	−2.2 (±16.1) −0.05 (9.1; 7.6)	0.515^b^	59.3 (±9.2) 58.3 (53.3; 67.1)	39.5 (±21.8) 40.1 (23.8; 58.8)	−19.9 (±18.8) −15.8 (−34.2; −3.1)	*p* < 0.001^b^
VAS (Range: 0−10)	7.6 (±1.6) 8.0 (7.0; 8.8)	7.6 (±1.9) 8.0 (7.0; 9.0)	−0.04 (±2.1) 0.0 (−1.0; 1.0)	0.935^a^	7.7 (±1.4) 8.0 (7.0; 9.0)	5.4 (±2.3) 6.0 (3.8; 7.3)	−2.3 (±2.5) −2.5 (−4.3; −0.8)	0.001^a^
VAS GI (Range: 0−10)	3.1 (±1.4) 3.0 (1.9; 4.7)	2.3 (±1.3) 2.2 (1.5; 2.6)	−0.9 (±1.3) −0.5 (−1.7; 1.7)	0.007^a^	3.4 (±1.5) 3.4 (2.2; 4.4)	1.4 (±1.3) 1.2 (0.1; 2.6)	−2.0 (±0.9) −2.1 (−2.7; −1.3)	*p* < 0.001^a^
BPI (Range: 0−20)	14.1 (±2.2) 14.4 (12.9; 15.2)	13.0 (±3.6) 13.4 (11.1; 15.5)	−1.1 (±2.7) −1.0 (−2.4; 1.1)	0.062^b^	12.5 (±2.3) 12.8 (10.8; 14.1)	8.7 (±4.7) 10.2 (4.4; 12.2)	−3.8 (±4.1) −3.2 (−5.7; −0.7)	*p* < 0.001^b^
PSQI (Range: 0−21)	15.1 (±4.0) 16.0 (12.0; 18.0)	13.9 (±4.5) 14.5 (11.0; 17.0)	−1.2 (±2.6) −1.0 (−2.8; 0.8)	0.037^b^	15.0 (±5.2) 15.0 (10.8; 19.5)	11.6 (±5.7) 9.5 (8.5; 16.3)	−3.5 (±4.6) −3.0 (−8.0; 0.8)	0.002^b^
FSS (Range: 0−7)	6.4 (±0.7) 7.0 (6.0; 7.0)	5.9 (±1.2) 6.0 (5.0; 7.0)	−0.5 (±1.0) 0.0 (−1.0; 0.0)	0.038^a^	5.5 (±1.1) 6.0 (4.8; 6.0)	4.4 (±1.7) 5.0 (3.8; 5.3)	−1.1 (±1.2) −1.0 (−2.0; 0.0)	0.001^a^
SF36 (Range: 0−100)	38.6 (±7.2) 38.9 (33.1; 42.7)	42.2 (±9.7) 42.2 (36.5; 47.2)	3.6 (±10.4) 2.2 (−4.9; 11.9)	0.137^a^	44.0 (±10.3) 42.6 (36.9; 53.5)	54.3 (±12.3) 58.4 (43.5; 63.6)	10.2 (±11.2) 9.0 (3.4; 15.9)	*p* < 0.001^a^
SF36 Physical Component (Range: 0−100)	30.9 (±8.2) 31.8 (22.6; 35.6)	34.8 (±14.3) 30.9 (21.7; 49.6)	3.9 (±13.5) 1.3 (−4.8; 13.6)	0.168^b^	33.4 (±11.4) 34.6 (25.0; 41.0)	51.5 (±18.8) 56.3 (36.5; 66.7)	18.1 (±20.0) 22.5 (−1.0; 36.0)	*p* < 0.001^b^
SF36 Mental Component (Range: 0−100)	38.6 (±15.8) 36.2 (26.2; 48.7)	47.2 (±19.8) 43.3 (28.9; 64.1)	8.5 (±23.1) 7.1 (−2.6; 24.7)	0.052^a^	54.4 (±23.1) 56.3 (33.7; 71.4)	63.4 (±21.4) 68.5 (51.3; 78.8)	8.9 (±21.0) 8.1 (1.9; 19.2)	0.023^a^

^1^Female participants.

FIQR, Revised Fibromyalgia Impact Questionnaire; VAS, Visual Analog Pain Scale; VAS GI, Visual Analog Scale from gastrointestinal symptoms; BPI, Brief Pain Inventory; PSQI, Pittsburg Sleep Quality Index; FSS, Fatigue Severity Survey; SF36, Short Form 36.

^a^p-value calculated by Wilcoxon Test between baseline and post-intervention, within-groups mean values;

^b^p-value calculated by Paired Samples t-test between baseline and post-intervention, within-groups mean values.

**TABLE 5:** Between-group analysis of clinical features.

Outcomes	Between-group analysis		Between-group post-intervention–baseline difference analysis *p*-value
	
	Baseline *p*-value	Post-intervention *p*-value	
FIQR (Range: 0−100)	0.676^a^	0.004^a^	0.001^b^
VAS (Range: 0−10)	0.937^a^	0.001^a^	0.002^b^
VAS GI (Range: 0−10)	0.660^a^	0.023^a^	0.002^b^
BPI (Range: 0−20)	0.015^a^	0.001^a^	0.011^b^
PSQI (Range: 0−21)	0.808^a^	0.073^a^	0.048^b^
FSS (Range: 0−7)	0.003^a^	0.001^a^	0.042^a^
SF36 (Range: 0−100)	0.047^a^	0.001^a^	0.045^a^
SF36 Physical Component (Range: 0−100)	0.454^a^	0.002^a^	0.008^b^
SF36 Mental Component (Range: 0−100)	0.015^a^	0.016^a^	0.947^b^

FIQR, Revised Fibromyalgia Impact Questionnaire; VAS, Visual Analog Pain Scale; VAS GI, Visual Analog Scale from gastrointestinal symptoms; BPI, Brief Pain Inventory; PSQI, Pittsburg Sleep Quality Index; FSS, Fatigue Severity Survey; SF36, Short Form 36.

^a^p-value calculated by Mann−Whitney between control and intervention groups mean values;

^b^p-value calculated by t-test for independent samples, between control and intervention mean values.

**FIGURE 1:**
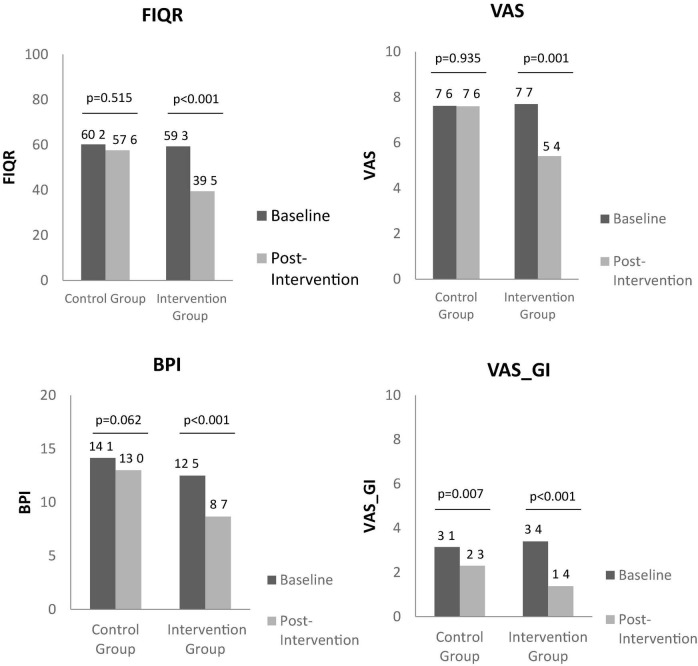
Between group baseline and post intervention analysis on the impact of fibromyalgia on quality of life, pain, and gastrointestinal symptoms, through FIQR, VAS, BPI, and VAS_GI, respectively. FIQR, Revised Fibromyalgia Impact Questionnaire; VAS, Visual Analogic Scale; BPI, Brief Pain Inventory; VAS GI, Visual Analog Scale from gastrointestinal symptoms.

**FIGURE 2:**
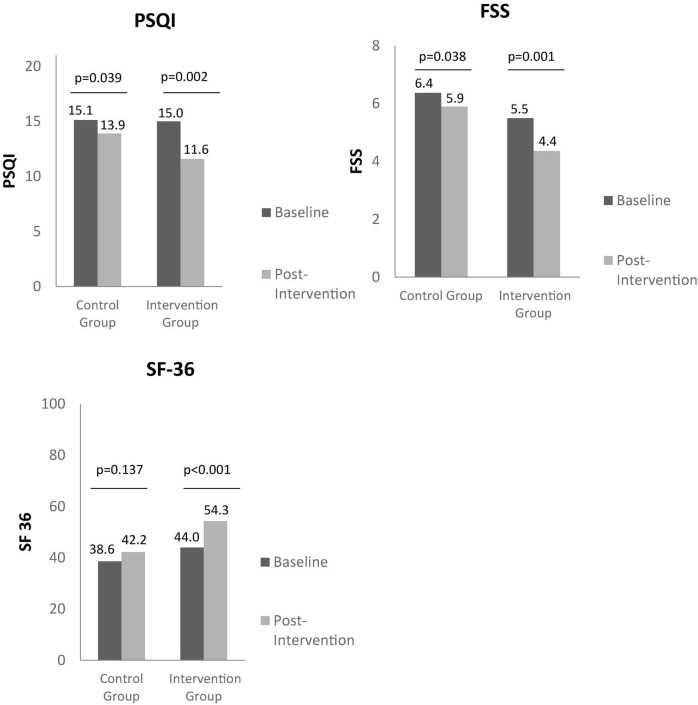
Between group baseline and post intervention analysis on sleep quality, fatigue, and quality of life, through PSQI, FSS, and SF36, respectively. PSQI, Pittsburg Sleep Quality Index; FSS, Fatigue Severity Survey; SF36, Short Form 36.

Through the observation of Figures 1, 2, it is possible to identify the differences between baseline and post-intervention, in the control group and intervention group.

At baseline, the between-group analysis showed no differences for the majority of parameters evaluated except for BPI, FSS, and SF36, for which the intervention group had more favorable baseline values.

In respect to intervention group, there was observed an improvement between baseline and post-intervention in FIQR (59.3 ± 9.2 vs. 39.5 ± 21.8; *p* < 0.001), in VAS (7.7 ± 1.4 vs. 5.4 ± 2.3; *p* = 0.001), BPI (12.5 ± 2.3 vs. 8.7 ± 4.7; *p* < 0.001), FSS (5.5 ± 1.1 vs. 4.4 ± 1.7; *p* = 0.001), VAS_GI (3.4 ± 1.5 vs. 1.4 ± 1.3; *p* < 0.001), PSQI (15.0 ± 5.2 vs. 11.6 ± 5.7; *p* = 0.002), SF36 (44.0 ± 10.3 vs. 54.3 ± 12.3; *p* < 0.001), SF36 physical component (33.4 ± 11.4 vs. 51.5 ± 18.8; *p* < 0.001), and SF36 mental component (54.4 ± 23.1 vs. 63.4 ± 21.4; *p* = 0.023).

In control group, there was also found an improvement in VAS_GI (3.1 ± 1.4 vs. 2.3 ± 1.3; *p* = 0.007), FSS (6.4 ± 0.7 vs. 5.9 ± 1.2; *p* = 0.038) and PSQI (15.1 ± 4.0 vs. 13.9 ± 4.5; *p* = 0.037) at the end of intervention compared to baseline.

Inflammatory biomarkers (hs-CRP, ESR) did not significantly change in both groups (Tables 6, 7).

**TABLE 6:** Biochemical parameters assessment in control and intervention group at baseline and post-intervention.

Outcomes	Control group (*n* = 24)^1^				Intervention group (*n* = 22)^1^			
		
	Baseline mean (±SD) median (P25;P75)	Post-intervention mean (±SD) median (P25;P75)	Post-intervention Δ mean (±SD) Δ median (P25; P75)	Within-group analysis *p*-value	Baseline mean (±SD) median (P25;P75)	Post-intervention mean (±SD) median (P25;P75)	Post-intervention Δ mean (±SD) Δ median (P25; P75)	Within-group analysis *p*-value
hs-CRP (mg/dL)	0.33 (±0.32) 0.24 (0.09; 0.43)	0.36 (±0.44) 0.23 (0.09; 0.49)	0.03 (±0.29) −0.03 (−0.15; 0.09)	0.920^a^	0.32 (±0.27) 0.21 (0.11; 0.53)	0.37 (±0.34) 0.19 (0.11; 0.62)	0.04 (±0.26) −0.0 (0.08; 0.15)	0.745^a^
ESR (mm)	10.42 (±8.20) 7.5 (5.0; 14.5)	9.88 (±8.83) 7.0 (5.0; 15.75)	−0.54 (±4.90) −0.5 (−3.0; 2.75)	0.663^a^	11.36 (±8.29) 8.0 (5.0; 14.25)	11.64 (±11.16) 8.50 (4.0; 13.75)	0.27 (±6.69) 0.0 (−4.3; 3.25)	0.794^a^

^1^Female participants.

hs-CRP, high-sensitive C-Reactive Protein; ESR, Erythrocyte Sedimentation Rate.

^a^p-value calculated by Wilcoxon Test between baseline and post-intervention, within-groups mean values;

^b^p-value calculated by Paired Samples t-test between baseline and post-intervention, within-groups mean values.

**TABLE 7:** Between-group analysis of biochemical parameters.

Outcomes	Between-group analysis		Between-group post-intervention variation analysis *p*-value
	
	Baseline *p*-value	Post-intervention *p*-value	
hs-CRP (mg/dL)	0.886^a^	0.750^a^	0.567^a^
ESR (mm)	0.650^a^	0.708^a^	0.640^a^

hs-CRP, high-sensitive C-Reactive Protein; ESR, Erythrocyte Sedimentation Rate.

^a^p-value calculated by Mann−Whitney between control and intervention groups mean values;

^b^p-value calculated by t-test for independent samples, between control and intervention mean values.

With regard to weight status and body composition, it was found that, in the control group, there were no differences between baseline and post-intervention (BMI: 29.5 ± 5.8 vs. 29.2 ± 5.5; *p* = 0.078; body fat percentage: 39.1 ± 8.9 vs. 37.7 ± 10.9; *p* = 0.181). However, in the intervention group, there were significant changes between the two moments, both in BMI (28.6 ± 4.1 vs. 27.6 ± 3.9, *p* > 0.001) and body fat percentage (38.5 ± 6.4 vs. 37.0 ± 7.0; *p* = 0.015).

It was possible to observe that, the impact of the intervention on FM symptoms was beneficial in the intervention group regardless of age, disease duration, BMI variation, and body fat mass variation between baseline and post-intervention. When the impact of the variation in the intake of each nutrient *per se* (monosaccharides, disaccharides, dietary fiber, omega 3 fatty acids, and omega 6 fatty acids) on FM clinical features was tested, there were no significant differences between post-intervention and baseline moments.

In the univariate analysis, there were no statistically significant associations between the patients’ reported outcomes and IMC variation, or monosaccharides and disaccharides, dietary fiber, Omega 3, and Omega 6 variation. The effect of the intervention between groups remains significant for FIQR, VAS, and VAS_GI after multivariate analysis.

## Discussion

After the anti-inflammatory and low FODMAPs nutritional intervention, there was an improvement in FM symptoms, namely pain, fatigue, gastrointestinal symptoms, quality of sleep, and quality of life in the intervention group.

Our results are aligned with other dietary interventions. An aspartame-free diet (56), a vegetarian diet (57, 58), and a hypocaloric diet (59, 60) reduced pain in patients with FM. Also, Marum et al. (42) found that a 4-week low FODMAPs diet reduced pain and improved quality of life in patients with FM. However, the dietary interventions carried out so far were of poor statistical quality, according to a recent systematic review (17).

Additionally, every study carried out so far tested the effect of isolated dietary strategies. In the present study, we used an integrative dietary approach, which included anti-inflammatory components and excluded the pro-inflammatory ones, therefore promoting more consistent results.

Fibromyalgia is characterized by a wide range of symptoms, which suggests several possible mechanisms of action explicative of the disease. In addition to the unknowing etiology of the disease, the dietary interventions carried out so far do not allow determining the best nutritional approach. However, several clinical trials have obtained positive results in relieving the symptoms of patients with FM, with different dietary interventions. In this sense, it is very likely that a multifactorial strategy would be the best way to approach this pathology.

The low FODMAPs protocol, given its high nutritional restriction, must be supervised by a specialist and applied for a short period of time, in order to avoid possible nutritional deficits. Although Gibson and Shepherd suggested 6−8 weeks of low FODMAPs diet application (16), other authors more recently have advocated that 4−6 weeks would be sufficient for a reduction in SIBO and associated gastrointestinal symptoms (11, 61). The application of this diet in FM patients, previously corroborated by another research team (42), aimed to alleviate gastrointestinal symptoms. Subsequently, the reintroduction of all excluded vegetables and fruits made it possible to increase fructooligosaccharides keeping the gastrointestinal function also healthy, as it was possible to verify through the respective questionnaire applied at the end of the study, the VAS_GI.

Additionally, the three months anti-inflammatory diet, rich in antioxidant compounds and Omega 3, and low in potentially inflammation-promoting foods, aimed to reduce the possible low-grade inflammation present in these patients, previously identified by several authors (62, 63), relieving especially pain and fatigue.

Our results provide a novel dietary intervention approach that combines dietary strategies with anti-inflammatory potential. We sought to study the impact of the overall dietary strategy, and not just isolated nutrients. Several authors defend that the effect of the overall diet or a dietary pattern appears to have more impact on chronic disease risk than looking for isolated nutrients (22, 23). This is due to the complex interactions and cumulative relationship between nutrients, along with the fact that nutrients do not target one particular tissue when consumed. Additionally, nutrients do not act in one specific metabolic pathway, or alone, which implies an important synergy between them (23). Dietary patterns allow more consistent results regarding the impact of different nutrients and foods on an individual’s health.

The results obtained in the statistical analysis concerning the identification of confounding variables, namely the absence of individual significant nutritional predictors, such as monosaccharides, disaccharides, dietary fiber, omega-3 fatty acids, and omega-6 fatty acids, reflect that the interventions with a traditional dietary approach, focusing on single nutritional factors, may not be enough to improve FM symptoms. To the best of our knowledge, this is the first study that brings together the multiplicity of food characteristics and nutritional factors with plausibility to improve FM symptoms.

In addition, our study considered a wide variety of outcomes, assessed through validated instruments, in order to broaden the ability to assess typical FM symptoms. We consider this aspect of great importance, given the broad spectrum of symptoms characteristic of the disease, and the absence of specific instruments for its assessment.

With respect to pharmacological therapy, patients were using analgesics, muscle relaxants, and anxiolytics, which were not expected to cause alteration in the intestinal microbiota (64).

It has been reported that weight loss was the main reason for pain improvement in FM patients in dietary interventions (65). However, in this study, we showed that the improvement in FM symptoms after intervention was independent of body fat mass percentage variation and BMI variation between baseline and post-intervention. This fact suggests that a hypocaloric diet and weight management may not be enough to improve FM symptoms.

Although the FM pathophysiology is not known, it has been suggested that genetic predisposition and stressful life events may trigger central and peripheral nervous system mechanisms (66), which are related to neuro-inflammation. The central nervous system (CNS) activation, associated with an apparent dysfunction in ascending and descending neural pathways in these patients, lead to an increased response mediated by amplification of CNS signaling. On the other hand, peripheral nervous system (PNS) is responsible for activation of mediators of innate immunity, promoting the release of bradykinin, histamine, serotonin, tumor necrosis factor (TNF), cytokines and IL, which translate inflammatory response and neuro-inflammation (67). In this context, the anti-inflammatory dietary approach employed in the present study may have contributed to reducing the systemic inflammatory process present in FM, and could provide an explanation of the mechanisms behind our findings. We also suggest that anti-inflammatory dietary intervention could also allow a more attenuated immune response, with a possible decrease in IL and pro-inflammatory cytokines. Although its alteration has already been detected in FM patients (68, 69), CRP and ESR biomarkers, which were used in our study, may not be specific enough, and that could possibly be the reason why there were no differences in our study between baseline and post-intervention.

Additionally, some authors revealed an association between FM and intestinal inflammation (41, 70, 71), derived from an alteration of the intestinal microbiota, with consequent intestinal dysbiosis and SIBO (11, 12, 72). Dysbiosis and metabolic endotoxemia are associated with a westernized dietary pattern rich in ultra-processed products, trans-fatty acids, sugars, and refined flour, along with stress and physical inactivity (73, 74). As a consequence, bacteria overgrowth and the release of endotoxins, hydrogen sulfide, phenols, ammonia, and indoles, expose intestinal mucosa and the host to harmful effects (38, 74). The FODMAPs mechanism of action is linked to the stimulation of mechanoreceptors as a response to luminal distension from a combination of increased luminal water content from the osmotic effect, especially in the small intestine, and from the release of hydrogen and ammonia from the bacterial fermentation of saccharides. Such stimulation can lead to ascending messages that might be interpreted as abdominal pain or bloating; reflex responses to the diaphragm and anterior abdominal wall, leading to increased abdominal distension; and effects on motility with a potential change in bowel habits. Furthermore, there could occur an excessive production of short-chain fatty acids, which could lead to visceral sensitivity and high-amplitude propagated colonic contractions, thus accelerating intestinal transit (38). In this context, limiting the intake of the most fermentable carbohydrates may have potentially alleviated gastrointestinal symptoms, by reducing gas formation.

The first month of a low FODMAPs diet seems to possibly reduce SIBO and optimize intestinal microbiota, allowing a greater efficacy of the posterior anti-inflammatory approach, and possibly of the pharmacological therapy, that patient was already being subjected to. The possible reduction of low-grade inflammation may be the explanation for the symptom improvement experienced by the intervention group.

Although it was also observed an improvement in gastrointestinal symptoms, fatigue, and quality of sleep, in the control group, the magnitude of the improvement was lower when compared to the intervention group. These improvements in the control group may be explained by the positive impact of WHO recommendations. However, once FM is associated with low-grade inflammation, those dietary recommendations *per se* do not seem to be anti-inflammatory enough.

This study has some limitations. The lack of a blood test for a low-grade inflammation specific cytokine such as IL-8, which has been associated with FM by several authors (2, 3) makes it impossible to objectively determine the symptoms improvement mechanisms or to confirm the reduction in low-grade inflammation. Moreover, it would be useful to test for a specific intestinal inflammation biomarker, such as fecal calprotectin.

Similarly, the primary evaluation of the presence of SIBO through a lactulose breath test would allow for objectively applying a low FODMAPs diet, which would be important from a clinical practice point of view. In that scenario, it would be possible to individually determine the necessity of this protocol application, which is relevant due to its severe nutritional restriction.

Moreover, the absence of assessment at the end of the first month of intervention makes it impossible to objectively assess the impact of a low FODMAPs diet alone and the real need to carry it out in this context. This would be imperative to adjust the methods of study replication. In this context, the authors considered this aspect as the major limitation of the study, which would be crucial to understanding the action mechanism of the dietary interventions.

Nevertheless, the aim of this study was to evaluate the impact of an integrated dietary approach, consisting of two different dietary interventions. According to the literature review, the results of the effect of a “low FODMAPs” intervention on FM have already been published, by Marum et.al (42). In this study, the authors found an improvement in pain, fatigue, and gastrointestinal symptoms after 4 weeks of a low FODMAPs diet. In contrast, the effect of an “anti-inflammatory diet” intervention, neither the effect of an integrated dietary approach that could simultaneously improve intestinal microbiota and reduce possible low-grade inflammation, has never been performed before.

Additionally, a low FODMAPS diet, due to the nature of its restriction, reduces bacterial diversity. However, the reintroduction of all excluded vegetables and fruits made it possible to increase fructooligosaccharides (FOS) ingestion, expectedly keeping the gastrointestinal function healthy, as it was possible to observe through the respective questionnaire applied at the end of the study, the Visual Analog Scale from Gastrointestinal Symptoms (VAS_GI).

Some particularities of both dietary strategies applied, namely low FODMAPs and an anti-inflammatory diet, may crossover during the intervention, such as the exclusion of lactose-free dairy in low FODMAPs and dairies in an anti-inflammatory diet. Additionally, removing gluten also removes fructans, one of the FODMAPs sub-unit (75), which has been identified as a possible cause of gastrointestinal symptoms, namely irritable bowel syndrome (76, 77). In this context, it is not possible to infer the impact of gluten exclusion in the outcomes analyzed in the present study. Nevertheless, taking into account that gliadin, present in gluten, may trigger an immunological reaction of inflammatory character (24, 25), we suggest that the absence of gluten might have acted beneficially.

At baseline, the sample homogeneity regarding total energy intake, omega-3 fatty acids, and also for added sugars and ultra-processed product intake parameters was not guaranteed, which may also be considered a potential bias. However, at the end of the intervention, we found the opposite differences, indicating that the ingestion of Omega 3 was higher, and added sugar and ultra-processed foods were low (non-existent) in the intervention group. With respect to energy, despite the difference between groups at the beginning and the end of the intervention (Table 3), there are no differences within the group between the two moments, which means that the calorie intake was maintained throughout the intervention. Additionally, body mass index and fat mass percentage suffered no changes between the baseline and post-intervention (Table 1), which reinforces the idea that caloric intake remained constant in both groups.

The study included only female participants, and this could be a limitation in the interpretation of the results. However, since FM affects mainly women, it has been decided to recruit only women for the present study, in order to avoid bias in the statistical analysis, since the sample size was expected to be low. Additionally, blinding the operator was also not possible due to human and financial resource restrictions.

Finally, in order to enhance the study results, further studies should be employed to enlarge the sample size.

Nevertheless, the limitations of this study contribute to clarifying the impact of an integrated dietary approach, which allows to draw some conclusions on this topic and provides some future lines of investigation.

Despite the proposed dietary restrictions, the diet was well accepted and the compliance was very good. Additionally, the exclusion of gluten, sugar, ultra-processed products, and dairy products in the intervention group was confirmed at the end of the intervention. Therefore, the application of this dietary intervention in clinical practice seems to be practicable and could be an important supporting tool for medical therapy in FM.

The present study allows us to conclude that an anti-inflammatory and low FODMAPs diet improved clinical features in this sample of patients with FM, which may represent a relevant complement to pharmacological therapy. The application of this dietary intervention in clinical practice, with the possibility of further personalization, should be encouraged.

## Data availability statement

The raw data supporting the conclusions of this article will be made available by the authors, without undue reservation.

## Ethics statement

The studies involving human participants were reviewed and approved by the Ethics Committee of Portuguese Institute of Rheumatology. The patients/participants provided their written informed consent to participate in this study.

## Author contributions

AS generated the random allocation sequence, enrolled the participants, assigned the participants to interventions, and conducted the intervention. JV-P, MS, and PP supervised the project. AS, AB, MS, and PP performed the statistical analysis. AS wrote the first version of the manuscript. All authors conceived and planned the study design, revised it critically for important intellectual content, provided the critical feedback, helped to shape the research and analysis, and have read and approved the final manuscript.

## Abbreviations

BPI: Brief Pain Inventory
FM: Fibromyalgia
FIQR: Fibromyalgia Impact Questionnaire Revised
FSS: Fatigue Severity Survey
PSQI: Pittsburg Sleep Quality Inventory
SF36: Short-form 36
SF36_Mental: Short-form 36 for Mental Aspect
SF36_Physical: Short-form 36 for Physical Aspect
VAS: Visual Analogic Pain Scale
VAS_GI: Visual Analogic Scale for Gastrointestinal Symptoms.
