# Supplementary material for: An anti-inflammatory and low fermentable oligo, di, and monosaccharides and polyols diet improved patient reported outcomes in fibromyalgia: A randomized controlled trial
Source: Front Nutr. 2022 Aug 15;9:856216. doi: 10.3389/fnut.2022.856216 (PMC9450131; doi:10.3389/fnut.2022.856216)
Supplement: Supplementary file 1 [file Data_Sheet_1.zip › Supplementary Material/Supplementary File 1.docx]

**Supplementary Figure S1. CONSORT diagram of the study.**

Analysed (n=22)

- Questionnaires applying: FIQR, SF-36, VAS, VAS-GI, BPI, PSQI, FSS
- Serum dosing: hs-CRP and ESR

Lost to follow-up:

- Stopped answering telephone contacts (n=4);
- Felt difficulty in compliance (n=3);
- No longer met inclusion criteria:
- Suffered a stroke (n=1);
- Suffered severe depression (n=1);
- AINEs injections because of sciatic nerve inflammation (n=1);

Lost to follow-up:

- Stopped answering telephone contacts (n=2);
- Emigrated to Switzerland (n=1);
- No longer met inclusion criteria:
- Corticoids injection because of back pain (n=1)
- Antibiotics because of urinary infection (n=1)

Analysed (n=24)

- Questionnaires applying: FIQR, SF-36, VAS, VAS-GI, BPI, PSQI, FSS
- Serum dosing: hs-CRP and ESR

Analysis

Follow-Up

Assessed for eligibility (n=62)

Excluded (n=1)

♦  Declined to participate (n=1)

Allocated to intervention group:

Anti-inflammatory and low FODMAPs diet (n=32)

Allocated to control group:

Healthy diet (n=29)

Allocation

Randomized (n=61)

Enrollment
