# Supplementary material for: An anti-inflammatory and low fermentable oligo, di, and monosaccharides and polyols diet improved patient reported outcomes in fibromyalgia: A randomized controlled trial
Source: Front Nutr. 2022 Aug 15;9:856216. doi: 10.3389/fnut.2022.856216 (PMC9450131; doi:10.3389/fnut.2022.856216)
Supplement: Supplementary file 1 [file Data_Sheet_1.zip › Supplementary Material/Supplementary File 2.docx]

Supplement 2 - Food alternatives poor in FODMAPs - adapted from Hill et. al., 2017 [[156](#_ENREF_156)].

| FODMAPs | Foods high in FODMAPs | Suitable alternatives low in FODMAPs |
| --- | --- | --- |
| Excess of Fructose | Fruits: apple, peach, mango, pear, pea, watermelon, preserves  Honey sweeteners: fructose, corn syrup  Large total dose of fructose: concentrated sources of fruit,  large portions of fruit, dried fruit, fruit juice | Fruits: banana, melon, grape, grapefruit, melon, kiwi, lemon, lime, orange, passion fruit, papaya,  raspberry, blueberry, strawberry, pineapple  Honey substitutes: maple syrup  Sweeteners: any sweeteners, except polyols |
| Lactose | Milk: regular and low-fat cow, goat, and sheep milk; ice cream  Yogurts: regular and low-fat yogurts  Cheeses: soft and fresh cheeses | Milk: lactose-free milk, rice milk  Ice cream substitutes: gelato, sorbet  Yogurts: lactose-free yogurts  Cheeses: hard cheeses |
| Oligosaccharides  (fructans and/or  galactans) | Vegetables: artichoke, asparagus, beet, broccoli,  Brussels sprouts, cabbage, fennel, garlic, leeks, okra,  onion, pea, shallot  Cereals: rye and wheat cereals (for example, biscuit, bread, couscous, biscuit, pasta)  Legumes: baked beans, chickpeas, lentils, red beans  Fruit: watermelon | Vegetables: bamboo root, spinach, carrot, celery, pak choy cabbage, cucumber, chives, corn, eggplant, green beans, lettuce, pumpkin, chard  Cereals: bread / cereals gluten-free and spelled  products  Fruit: tomato |
| Polyols | Fruits: apple, apricot, avocado, cherry, lychee, nectarine, peach, pear, plum, watermelon  Vegetables: cauliflower, mushroom, pea  Sweeteners: isomalt, maltitol, mannitol, sorbitol, xylitol, and other sweeteners ending in "-ol" | Fruits: banana, blueberry, melon, grape, grapefruit, melon, kiwi, lemon, lime, orange, passion fruit, papaya, raspberry  Sweeteners: glucose, sugar (sucrose), other artificial sweeteners that do not end in "-ol" |
