# Supplementary material for: An anti-inflammatory and low fermentable oligo, di, and monosaccharides and polyols diet improved patient reported outcomes in fibromyalgia: A randomized controlled trial
Source: Front Nutr. 2022 Aug 15;9:856216. doi: 10.3389/fnut.2022.856216 (PMC9450131; doi:10.3389/fnut.2022.856216)
Supplement: Supplementary file 1 [file Data_Sheet_1.zip › Supplementary Material/Supplementary File 3.docx]

Supplement 3 – Low FODMAPs week diet example

|  | **Monday** | **Tuesday** | **Wednesday** | **Thursday** | **Friday** |
| --- | --- | --- | --- | --- | --- |
| **Breakfast** | Oatmeal porridge with water, lime and cinnamon | 2 scramble eggs  + 1 orange | 5 rice coockies (only rice, water and salt)  + strawberry jam  + tea, sugar free | Oatmeal porridge with water, lime and cinnamon | 2 scramble eggs  + 1 orange |
| **Snack** | 1 banana | 2 kiwis | 1 orange | 2 kiwis | 2 kiwis |
| **Lunch** | Chicken steak, grilled, with rice and salad (lettuce and tomato) with olive oil;  Water | White rice and tuna fish, with tomato and cucumber;  Water | Grilled salmon with lemon juice, rice and salad (lettuce and tomato);  Water | Turkey steak, grilled, with rice and salad (lettuce and tomato) with olive oil;  Water | Grilled chicken, shrimp and pineapple skewers, with rice and salad (lettuce and tomato);  Water |
| **Snack** | 2 boiled egg  + 5 strawberries | 1 microwave cake: 1 banana, 1 egg, 3 tablespoon of oat, cinnamon | 1 Pancake: 1 banana, 1 egg, 2 tablespoon of oat  + 5 strawberries | 1 banana  + 5 rice coockies (only rice, water and salt) | 5 rice coockies (only rice, water and salt)  + strawberry jam |
| **Dinner** | Soap: potato, carrot, cucumber, spinach, coriander;  Roasted red fish with roasted potatos;  Water | Grilled sardines with boiled potato and salad (lettuce and tomato);  Water | Salad (lettuce, tomato, carrot, spinach), tuna fish, boiled egg, almonds and seeds; olive oil;  Water | Omelet with tomato and a salad (lettuce and tomato) with olive oil;  Water | Whitefish with lime, boiled potato and green beans;  Water |
